# Hypoxic stress increases NF-κB and iNOS mRNA expression in normal, but not in keratoconus corneal fibroblasts

**DOI:** 10.1007/s00417-020-04900-8

**Published:** 2020-09-04

**Authors:** Tanja Stachon, Lorenz Latta, Berthold Seitz, Nóra Szentmáry

**Affiliations:** 1 Department of Ophthalmology, Saarland University Medical Center, Homburg, Saar Germany; 2 Dr. Rolf. M. Schwiete Center for Limbal Stem Cell and Aniridia Research, Saarland University, Homburg/Saar, Germany; 3 Department of Ophthalmology, Semmelweis University, Budapest, Hungary

**Keywords:** Keratoconus, Hypoxia, NF-κB, iNOS, HIF, PHD2, Inflammation, Pathogenesis

## Abstract

**Background:**

Keratoconus (KC) is associated with oxidative stress and hypoxia and as several times discussed, potentially with inflammatory components. Inflammation, hypoxia, and oxidative stress may result in metabolic dysfunction and are directly linked to each other. In the current study, we investigate the effect of hypoxia through NF-κB signaling pathways on iNOS, hypoxia-induced factors (HIF), ROS, and proliferation of normal and KC human corneal fibroblasts (HCFs), in vitro.

**Methods:**

Primary human KC-HCFs and normal HCFs were isolated and cultured in DMEM/Ham’s F12 medium supplemented with 5% fetal calf serum. Hypoxic conditions were generated and quantitative PCR and Western blot analysis were performed to examine NF-κB, iNOS, HIF, and PHD2 expression in KC and normal HCFs. ROS level was analyzed using flow cytometry and proliferation by BrdU-ELISA.

**Results:**

Hypoxia increased NF-κB mRNA and protein expression in normal HCFs, but in KC-HCFs NF-κB mRNA and protein expression remained unchanged. Hypoxic conditions upregulated iNOS mRNA expression of normal HCFs, but iNOS mRNA expression of KC-HCFs and iNOS protein expression of both HCF types remained unchanged. Hypoxia downregulated HIF-1α and HIF-2α mRNA expression in normal and KC-HCFs. PHD2 mRNA expression is upregulated under hypoxia in KC-HCFs, but not in normal HCFs. PHD2 protein expression was upregulated by hypoxia in both HCF types. Total ROS concentration is downregulated in normal and KC-HCFs under hypoxic conditions. Proliferation rate of KC-HCFs was upregulated through hypoxia, but did not change in normal HCFs.

**Conclusions:**

Hypoxia increases NF-κB and iNOS mRNA expression in normal HCFs, but there does not seem to be enough capacity in KC-HCFs to increase NF-κB and iNOS mRNA expression under hypoxia, maybe due to the preexisting oxidative stress. HIF and PHD2 do not show altered iNOS regulation under hypoxic conditions in KC-HCFs, and therefore do not seem to play a role in keratoconus pathogenesis. An increased proliferation of cells may refer to compensatory mechanisms under hypoxia in KC. Understanding the mechanism of the altered regulation of NF-κB and iNOS in KC-HCFs will provide better insight into the potential inflammatory component of the KC pathogenesis.
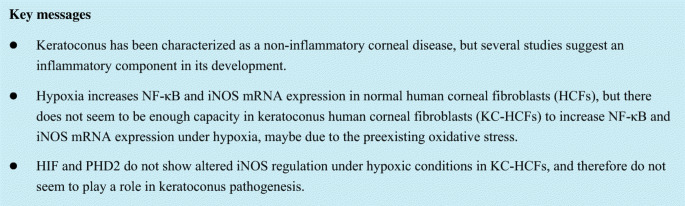

## Background

Keratoconus (KC) is a progressive corneal disease characterized by thinning and deformation, but with unknown etiology. So far, KC has been characterized as a non-inflammatory corneal disease, but several studies suggest an inflammatory component in its development [1, 2]. Karamichos et al. suggested the involvement of altered metabolic pathways in its pathogenesis, but it has also been described that oxidative stress plays a role [3–5].

It is known that reactive oxidative species (ROS) are elevated in KC corneas [5]. The reason could be, among others, altered metabolic pathways or hypoxic conditions due to mitochondrial dysfunction of mtDNA-mediated inflammation. If a hypoxic state is present due to altered metabolic functions, these also have an influence on the oxidative state and can act as a pro-inflammatory mediator. Both hypoxic conditions and oxidative stress trigger an inflammatory cell response [6].

In our previous study, we determined an increased inducible nitric oxide synthase (iNOS) and nuclear factor kappa B (NF-κB) mRNA expression in KC-HCFs compared with normal cells [7]. This was accompanied by an increased NF-κB protein expression, without a change in iNOS protein levels.

**Fig. 1:**
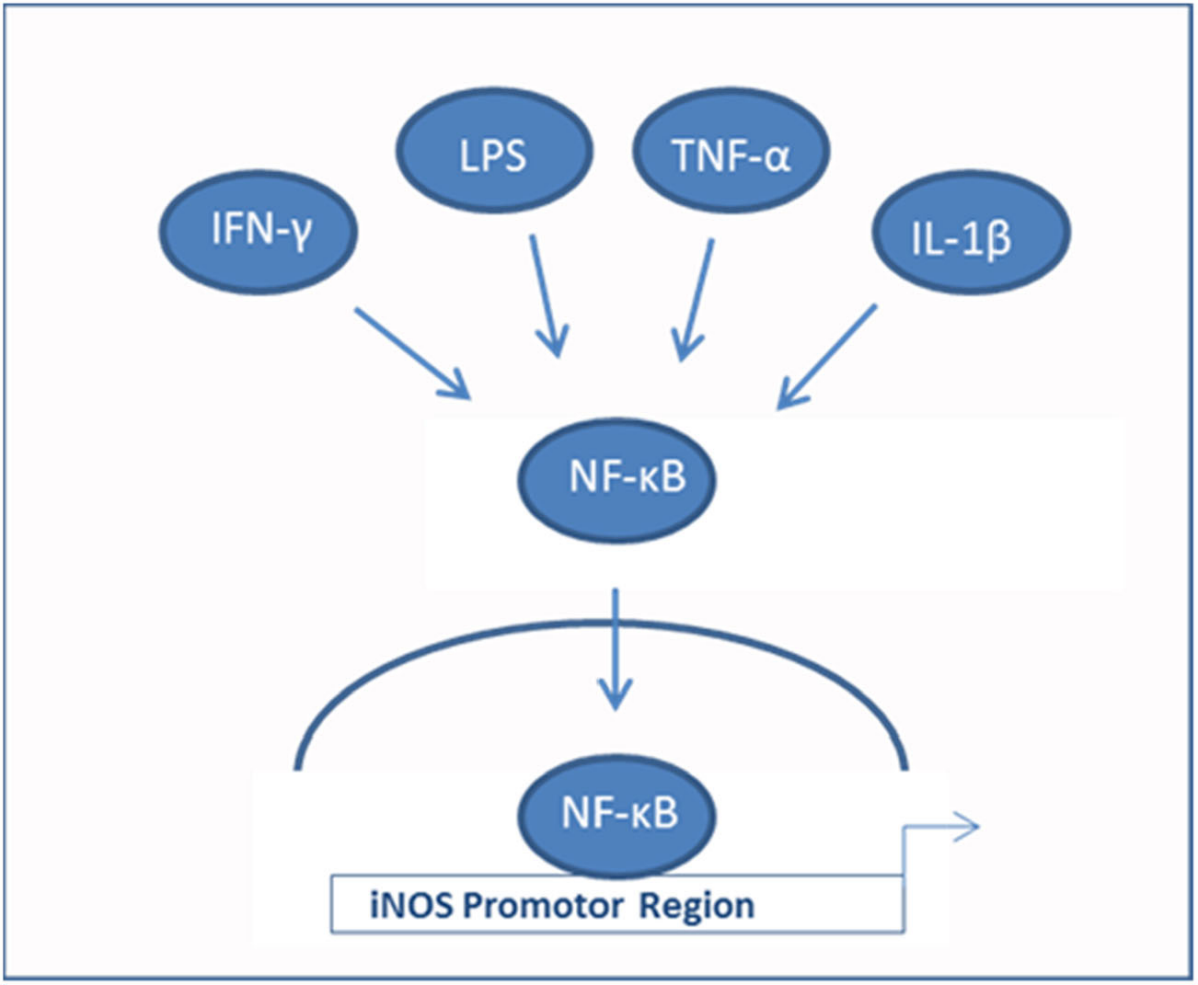
Activation of iNOS transcription through NF-κB binding at the iNOS promotor region. The scheme displays the activation of NF-κB by IFN-γ, LPS, TNF-α, or IL-1β. NF-κB can directly bind to the iNOS promotor region to activate the transcription of iNOS which is able to start the production of nitric oxide

iNOS converts L-arginine into nitric oxide (NO), which reacts as a free radical and is also part of the ROS/RNS state in the cells. The induction of iNOS is mainly mediated by inflammatory cytokines such as IL-1, TNF-α, and IFN-γ, but also by lipopolysaccharides (LPS) in macrophages via NF-κB (Fig. 1). However, several cell types constitutively express iNOS without an inflammatory process [8]. It is also known that ROS production by KC-HCFs is higher than that in HCFs.

**Fig. 2:**
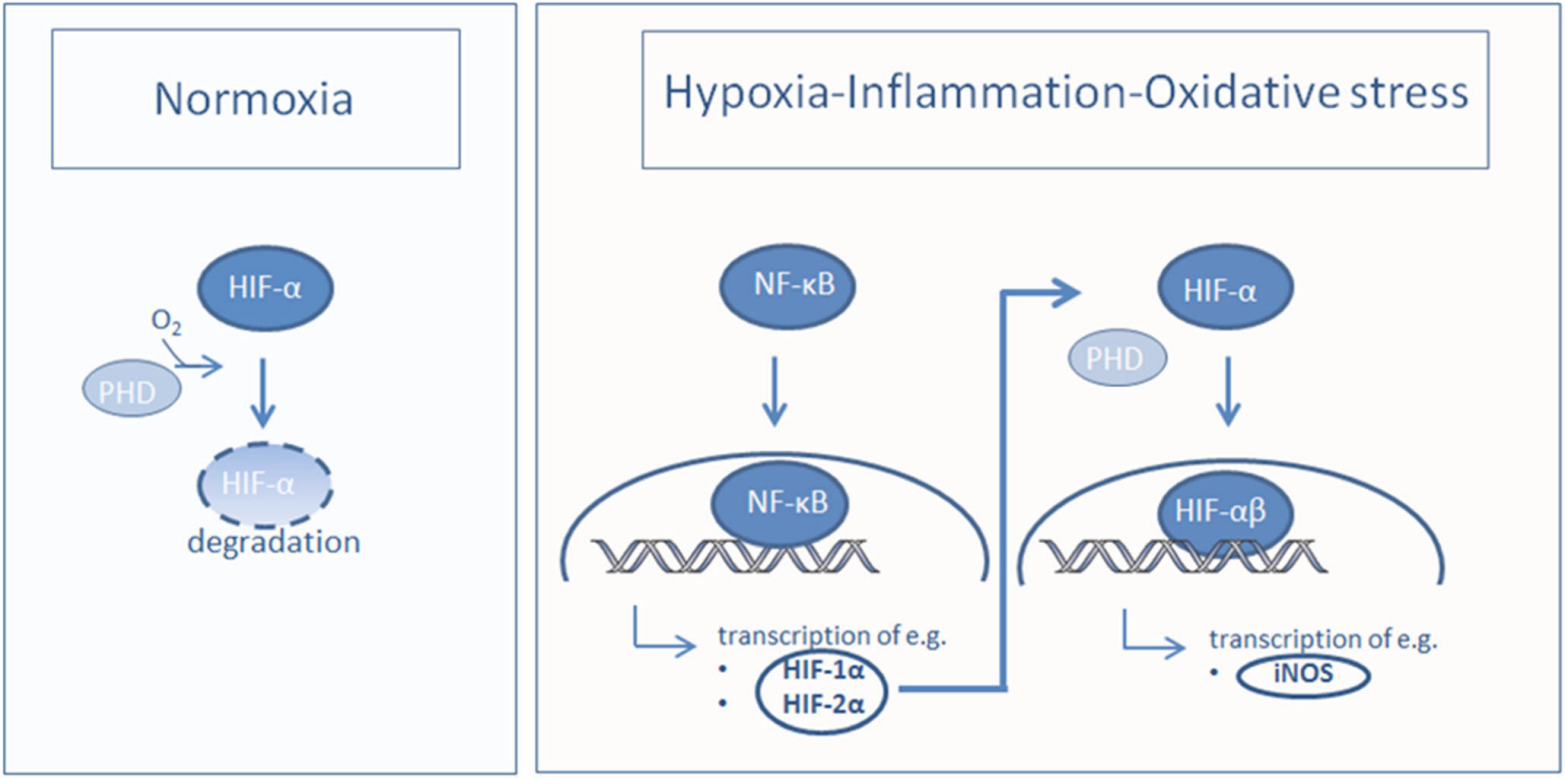
Under normoxia, HIF-α is hydroxylated by PHDs, which target HIF-α for von Hippel - Lindau protein degradation. Under hypoxic conditions, through inflammation or oxidative stress, HIF-α transcription is induced via NF-κ dependent mechanism. HIF-α is located in the cell nucleus and binds to the β unit and serves as a transcription factor, e.g. for iNOS.

The hypoxia-inducible factor α (HIFα) acts as an oxygen sensor in the cellular response to reduced oxygen supply [9]. Its main subunits are HIF-1α and HIF-2α. Under hypoxic conditions, HIFα is stabilized by prolyl-hydroxylases (PHDs) and translocated into the cell nucleus. Then, the α subunit forms a complex with HIFβ and acts as a transcription factor in the hypoxic reaction, e.g., for iNOS (Fig. 2).

In keratoconus, cell density is reduced in all layers of the cornea [10]. However, hypoxia can induce proliferation of many cell types and is known as a regulatory factor [11]. All factors, ROS/RNS and oxygen homeostasis via HIF-1α and PHDs and proliferation, are involved in the interaction of inflammation and altered metabolic balance and influence each other.

In this study, we investigated the regulation of NF-κB, iNOS, HIF-1α, HIF-2α, and PHD expression; ROS, and proliferation under hypoxic conditions in keratoconus corneal fibroblasts compared with normal control cells, in vitro.

## Methods

### Cell culture

Eight normal human corneas were obtained from the LIONS Cornea Bank Saar-Lor-Lux, Trier/Westpfalz, and 8 corneas from KC patients were obtained from elective penetrating keratoplasties. For the keratoconus corneas, all eyes with previous ocular surgery have been excluded. Donor corneas, which did not match the criteria for transplantation (less than 2000 endothelial cells/mm^2^), have been used for the experiments.

To isolate keratocytes, human corneoscleral/corneal buttons were first rinsed in PBS (Merck Sigma-Aldrich Chemie GmbH, Taufkirchen, Deutschland). The central corneal button was cut into small pieces and was incubated in culture medium with 1.0 mg/ml collagenase A (Roche Diagnostic GmbH, Mannheim, Germany, No. 10103578001) for 24 h at 37 °C. The digested tissue and cells were centrifuged at 800 *g* for 7 min and resuspended in culture medium, which consisted of basic medium (DMEM/F12, Sigma-Aldrich® GmbH, Geisenheim, Germany, No. 11594426) supplemented by 5% fetal calf serum (FCS) (Fisher Scientific GmbH, Schwerte, Germany, No. 11573397). The cell suspension was seeded in 75-cm^2^ culture flasks, using DMEM/F12 (+ 5% FCS). Thereafter, medium was changed every 2 to 3 days until cells reached confluence. As keratocytes were cultured with FCS, we specify them further on in the manuscript as “human corneal fibroblasts” (HCF) or “keratoconus human corneal fibroblast” (KC-HCF) [12].

The cell passages 3 to 8 were used for the experiments.

Hypoxic conditions were generated using 150 μM CoCl_2_ (PHD inhibitor, Sigma-Aldrich® GmbH, Geisenheim, Germany, No. 60818) for 4 h, 24 h, and 48 h, to evaluate the time-point with changes in NF-κB, iNOS, HIF-1α, and HIF-2α mRNA and protein expression. Since CoCl_2_ is an inhibitor of prolyl-hydroxylases, which induce a chemical hypoxia, we used 1% O_2_ atmosphere in a hypoxic chamber (Modular Incubator Chamber, Billups-Rothenberg, Inc., CA, USA) to examine the effect of hypoxia on prolyl-hydroxylase 2 [13]. For PHD2 analysis, we generated 1% O_2_ for 48 h for PHD2 mRNA and protein expression measurements. Shortly, the cell culture flask was placed in a hypoxic chamber; the chamber was connected to a gas mixture (1% O_2_, 5% Co_2_, 94% N_2_) and was filled with the gas for 2 min. This procedure was repeated after 24 h. The chamber including the cell culture flasks was incubated at 37 °C in the cell culture incubator.

### RNA isolation and cDNA synthesis

Human normal and KC-HCFs were seeded in 75-cm^2^ cell culture flasks. After reaching confluence, the cells were harvested using Trypsin-EDTA (0.05% trypsin/0.02% EDTA, Sigma-Aldrich® GmbH, Geisenheim, Germany). The RNA isolation was performed according to the manufacturer’s protocol (ISOLATE II RNA/DNA/Protein Kit, Luckenwalde, Germany) and RNA was stored at − 80 °C until cDNA synthesis (One Taq® RT-PCR Kit, New England Biolabs INC, Frankfurt, Germany). For cDNA, 1 μg of total RNA was used as template for all samples. The cDNA was stored at − 20 °C until further use.

### Quantitative PCR

For quantitative PCR (qPCR), Tata-binding protein (TBP), NF-κB (RELA/NF-κB p65), HIF-1α, HIF-2α, and PHD2 validated primer sets for use in SYBR Green–based quantitative PCR, obtained from Qiagen GmbH (Hilden, Germany), were utilized. For iNOS, primers were synthesized by MWG Eurofins (Table 1).

**Table 1:** Primer pairs for qPCR

Targeted cDNA	Gene symbol	5′ forward primer 3′ or Qiagen Cat. No.	Amplicon size bp
NF-κB	RELA	QT02324308	136
iNOS Forward 5′ → 3′ Reverse 5′ → 3′	NOS2	CTGGCAAGCCCAAGGTCTAT GGAGGCTCCGATCAATCCAG	517
HIF-1α	HIF1A	QT00083664	104
HIF-2α	EPAS1	QT00069587	127
HIF-PHD2	EGLN1	QT00222684	123
P4H-TM	P4HTM	QT00041048	87
TBP	TBP	QT00000721	132

The qPCR experiment was carried out for all KC samples and for controls in 96-well plates using AceQ SYBR qPCR Master Mix (Vazyme Biotech, China) and a PCR Thermocycler QuantStudio 5 Real-Time PCR System (ThermoFisher Scientific™ GmbH, Dreieich, Germany).

The relative normalized expression of NF-κB, iNOS, HIF-1α, HIF-2α, and PHD2 was compared with the respective TBP reference gene. The ΔΔ cycle threshold (Ct) fold change was quantified by comparing the Ct obtained from the unknown samples compared with the Ct of the reference gene TBP. For qPCR, the amplification conditions were 95 °C for 10 s, 64 °C for 10 s, and 72 °C for 45 s and 40 cycles.

### Western blot analysis

To determine NF-κB, iNOS, HIF-1α, HIF-2α, and PHD2 protein expression in corneal fibroblasts, 20 μg protein of normal or KC human corneal fibroblasts, obtained with ISOLATE II RNA/DNA/Protein Kit (Luckenwalde, Germany), was used (Western blot analysis). Antibodies against NF-κB p65, HIF-1α, HIF-2α, and PHD2 were purchased from Cell Signaling Technology (Frankfurt am Main, Germany), and anti-iNOS antibody from Abcam (Cambridge, UK). After boiling the samples for 5 min at 95 °C, proteins were separated using NuPAGE ™ bis-tris precast 4–12% bis-tris gels (ThermoFisher Scientific™ GmbH, Dreieich, Germany). Following protein separation, the proteins were transferred onto a nitrocellulose membrane with the Trans Blot Turbo Transfer System (BioRad, Hercules, CA, USA). Primary antibodies were diluted in WesternFroxx anti-rabbit HRP solution containing blocking reagent and secondary antibody (BioFroxx GmbH, Einhausen, Germany). For loading control, blots were stripped in stripping buffer (BioFroxx), and reprobed with calnexin antibody (Enzo Life Sciences Ag, Lausen, Switzerland, No. ADI-SPA-865). Visualization was performed using an imaging system (LAS 4000 system, Fujifilm).

### Protein measurement

For Western blot analysis, protein quantity was determined according to Bradford’s method, using bovine serum albumin as a standard. The absorbance was measured at 595 nm and the concentrations were quantified.

### ROS detection

To evaluate total ROS concentration in keratocytes, the Total ROS assay kit for flow cytometry was used (Thermo Fisher Scientific, Karlsruhe, Germany). Shortly, cells were seeded into 6-well plates and were cultivated until 90% confluence. One hundred fifty micrometers micromolar (150 µM) of the hypoxia-mimicking agent CoCl_2_ was used to generate hypoxic conditions for 48 h. Thereafter, cells were harvested and stained with ROS assay stain for 60 min at 37 °C. FACS analysis was performed using FACS Canto (Becton Dickinson, Heidelberg, Germany) of the 488 nm FITC channel. Ten thousand events were acquired for each analysis. Geometric mean of fluorescence intensity was evaluated using WinMDI 2.9.

### BrdU assay

Proliferation of HCFs was assessed after culturing the cells under hypoxic conditions using 150 μM CoCl_2_ with the proliferation ELISA-BrdU kit, by the measurement of BrdU (5-bromo-2′deoxyuridine) incorporation in the newly synthesized cellular DNA. HCFs were seeded in a 96-multiwell plate at a density of 6 × 10^3^ cells/cm^2^ in 100 μl culture medium per well. After a growth period of 24 h, the culture medium was changed to a CoCl_2_ containing culture medium, at 37 °C for 48 h. The test was performed according to the manufacturer’s protocol (Cell Proliferation ELISA, BrdU colorimetric, Roche, No 11647229001, Sigma-Aldrich® GmbH, Geisenheim, Germany).

### Statistical analysis

For statistical analysis, the GraphPad Prism 7.04 was used. Statistical analysis was performed using the Mann-Whitney *U* test for comparison between groups. *p* values below 0.05 were considered statistically significant.

## Results

### NF-κB, iNOS, HIF-1α, HIF-2α, and PHD2 mRNA expression and Western blot analysis under normoxic and hypoxic conditions

mRNA and Western blot measurements were performed following hypoxic conditions for 4, 24, and 48 h. Below we summarize results for the time-points with changes in mRNA and protein expression, which is also summarized at Table 2. For the other time-points, without changes in mRNA and protein expression, compared with controls (under hypoxic conditions) data are not shown below or at Table 2.

**Table 2:** Comparison of NF-κB, iNOS, HIF-1α, HIF-2α, PHD2 mRNA, and protein expression and total ROS concentration under normoxic and hypoxic conditions in normal HCFs and in KC-HCFs (Mann-Whitney *U* test). NF-κB mRNA is displayed after 4 h; iNOS, HIF-1α, and HIF-2α after 48 h; and PHD2 mRNA after 4 h and 48 h of hypoxic conditions. Hypoxic conditions for Western blot analysis of NF-κB iNOS, HIF-1α, HIF-2α, and PHD2 were 48 h (*p < 0.05; **p < 0.01; ***p < 0.001)

Parameters	Normoxic conditions	Normoxic vs hypoxic conditions (48 h)	
	Controls (HCFs) vs KC-HCFs	Normal HCFs	KC-HCFs
NF-κB mRNA expression	Increased in KC-HCFs**^7^	Increased**	Unchanged
NF-κB protein expression	Increased in KC-HCFs**^7^	Unchanged	Unchanged
iNOS mRNA expression	Increased in KC-HCFs***^7^	Increased*	Unchanged
iNOS protein expression	Unchanged	Unchanged	Unchanged
HIF-1α and HIF-2α mRNA expression	Unchanged	Decreased*	Decreased***
HIF-1α and HIF-2α protein expression	Unchanged	Unchanged	Unchanged
PHD2 mRNA expression	Increased in KC-HCFs (4 h)** Unchanged (48 h)	Increased (4 h)* Unchanged (48 h)	Decreased (4 h)* Increased (48 h)**
PHD2 protein expression	Unchanged	Increased*	Increased*
Total ROS concentration	Increased in KC-CFs**	Decreased**	Decreased*
Proliferation rate of HCFs	Decreased in KC-HCFs*	Unchanged	Increased**

No. 7 indicates the literature reference given in the bibliography

**Fig. 3:**
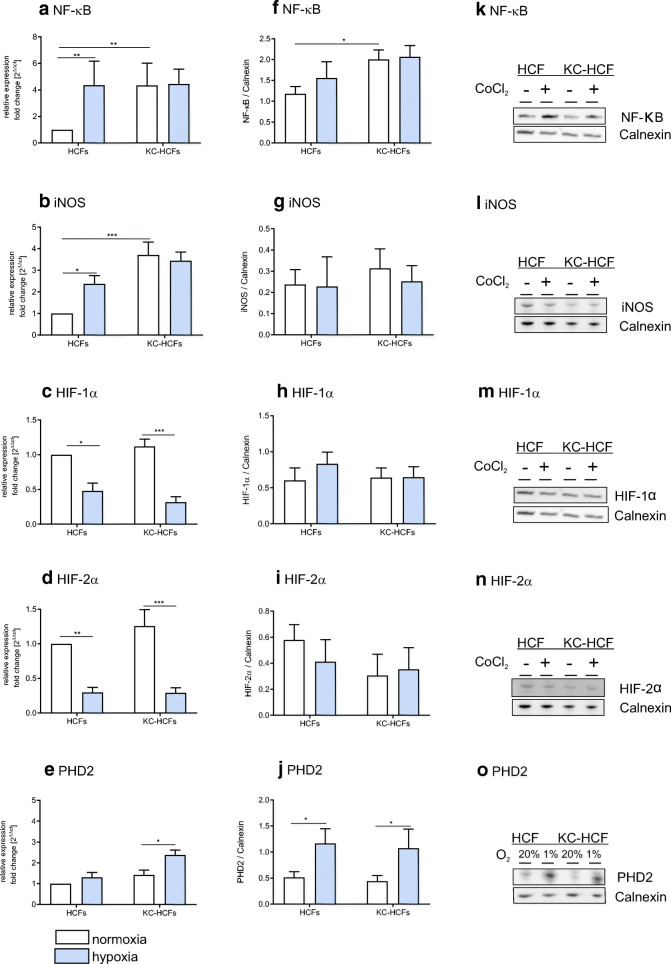
Effect of hypoxia on NF-κB, iNOS, HIF-1α, HIF-2α, and PHD2 mRNA and protein expression (normoxic conditions: white bars; hypoxic conditions: blue bars). Hypoxic conditions for human corneal fibroblasts (HCFs) and keratoconus human corneal fibroblasts (KC-HCFs) were performed using 150 μM CoCl_2_ for NF-κB (4 h), and iNOS, HIF-1α, and HIF-2α (48 h) for PHD2 1% O_2_ atmosphere were generated in a hypoxic chamber for 48 h. Significant differences are indicated. **a**–**e** Quantitative mRNA analysis of NF-κB, iNOS, HIF-1α, HIF-2α, and PHD2. Data show mean ± SEM of at least 8 independent experiments in duplicate. **f**–**j** Relative quantification of Western blot analysis. Calnexin was used as loading control and to calculate the relative protein expression levels. **k**–**o** Representative Western blots of NF-κB, iNOS, HIF-1α, HIF-2α, and PHD2 (*p < 0.05; **p < 0.01; ***p < 0.001)

Figure 3 displays **NF-κB, iNOS, HIF-1α, HIF-2α**, and **PHD2** mRNA and protein expression under normoxic and hypoxic conditions. NF-κB mRNA is displayed after 4 h and iNOS, HIF-1α, HIF-2α, and PHD2 mRNA after 48 h of hypoxic conditions. Hypoxic conditions for Western blot analysis of NF-κB iNOS, HIF-1α, HIF-2α, and PHD2 were 48 h.

In KC-HCFs, **NF-κB** mRNA (*p* = 0.0098) and protein (*p* = 0.0012) expression was increased compared with normal HCFs, under normoxic conditions. Through hypoxia for 4 h, **NF-κB** mRNA expression increased in normal HCFs (*p* = 0.0089), but **NF-κB** mRNA expression in KC-HCFs and **NF-κB** protein expression in normal and KC-HCFs remained unchanged (*p* > 0.721).

**iNOS** mRNA expression was significantly higher in KC-HCFs than in normal HCFs (*p* < 0.0001) under normoxic conditions, but iNOS protein expression did not differ between both HCF types (*p* = 0.841). Hypoxic conditions for 48 h significantly increased iNOS mRNA expression of normal HCFs (*p* = 0.044), but iNOS mRNA expression of KC-HCFs and iNOS protein expression of both HCF types did not change significantly through hypoxia (*p* > 0.421), compared with controls.

**HIF-1α** and **HIF-2α** mRNA and protein expression did not differ between both HCF types, under normoxic conditions (*p* > 0.469). Following hypoxic conditions for 48 h, **HIF-1α** and **HIF-2α** mRNA expression was decreased in normal (*p* = 0.014 for both) and KC-HCFs (*p* < 0.0001 for both), compared with controls, but **HIF-1α** and **HIF-2α** protein expression remained unchanged (*p* > 0.393).

Under normoxic conditions, **PHD2** mRNA and protein expression did not differ between normal and KC-HCFs (*p* > 0.558). Under hypoxic conditions for 4 h, **PHD2** mRNA expression increased in HCFs (*p* = 0.0069) and decreased in KC-HCFs (*p* = 0.0046) (data not shown at figures). Under hypoxic conditions for 48 h, **PHD2** mRNA expression remained unchanged in normals (*p* = 0.496), but increased in KC-HCFs (*p* = 0.0096). **PHD2** protein expression increased in both HCF types following 48 h of hypoxia (*p* = 0.031 for both).

### Total ROS concentration in KC and normal HCFs

**Fig. 4:**
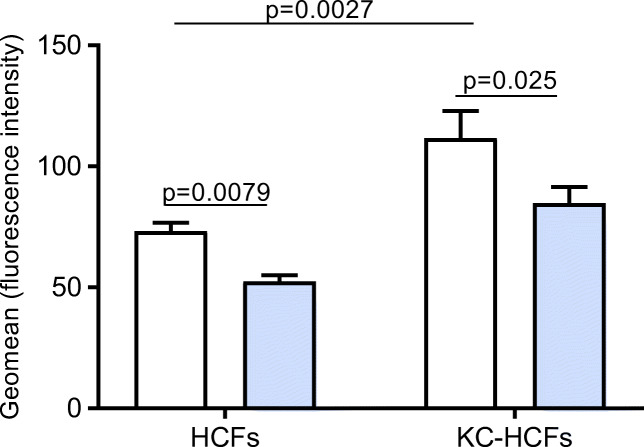
Total reactive oxygen species (ROS) concentration under normoxic (white bars) and hypoxic conditions (blue bars) using 150 μM CoCl_2_ for 48 h in normal and KC-HCFs. KC-HCFs show a higher ROS level than control cells. The concentration of ROS is reduced in both cell types following hypoxia. Data were presented as mean ± SEM of 8 independent experiments. Significant differences are indicated

Under normoxic conditions, total ROS concentration was significantly higher in KC-HCFs than in normal HCFs (*p* = 0.0027). Hypoxic conditions for 48 h resulted in decreased total ROS concentration in normal (*p* = 0.0079) and KC-HCFs (*p* = 0.025), compared with controls (Fig. 4).

### Proliferation of KC and normal HCFs

**Fig. 5:**
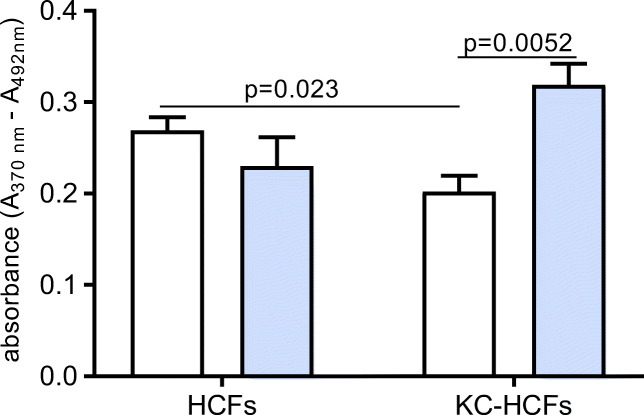
Proliferation under normoxic (white bars) and hypoxic conditions (blue bars) using 150 μM CoCl_2_ for 48 h in normal and KC-HCFs. The proliferation rate is reduced in KC-HCFs compared with normal cells. Hypoxia increased proliferation in KC-KCFs, but not in normal controls. Data were presented as mean ± SEM of 8 independent experiments. Significant differences are indicated

Under normoxic conditions, proliferation rate in KC-HCFs was lower as in normal HCFs (*p* = 0.023). Through hypoxia for 48 h, proliferation rate of KC-HCFs increased significantly (*p* = 0.0052), but did not change in normal HCFs (*p* = 0.244) (Fig. 5).

## Discussion

The pathogenesis of keratoconus is still unknown, but several changes in the stromal cells of keratoconus patients have been described. These include genetic, metabolic, and hormonal changes that are correlated with the disease [2, 14–18]. Some of these changes can be associated with increased ROS production in corneal fibroblasts of KC patients [3, 5].

KC is traditionally classified as a non-inflammatory corneal disease. However, in recent years, numerous study groups have demonstrated elevated pro-inflammatory cytokine concentrations in tear fluid of KC patients [19–23]. An inflammatory response is often associated with hypoxia and altered oxidative stress levels [6]. In our previous study, we described that the expression of iNOS mRNA in KC keratocytes is increased compared with normal cells [7], but without a detectable change in the iNOS protein. Normally, iNOS is expressed under inflammatory conditions via the NF-κB activation mechanism to produce more NO for the immune response [24]. However, there are also some cell types that permanently express iNOS. The function of constitutively expressed iNOS is not yet fully understood.

In this study, we investigated NF-κB, iNOS, HIF-1α, and HIF-2α as well as PHD2 expression, ROS production, and proliferation rate under hypoxic conditions in keratoconus corneal fibroblasts in vitro. Our study describes the altered regulation of KC-HCFs, compared with normal HCFs under hypoxic conditions. As the study was performed in cell culture cells, the growth and incubation conditions for the cells were optimal. Therefore, limitation of our study is that a comparison with “in vivo” is difficult.

Our results showed that the regulation of these parameters under hypoxic conditions is altered in KC-HCFs compared with normal corneal fibroblasts. NF-κB and iNOS mRNA expression is increased in KC-HCFs compared with HCFs. After induction of a hypoxic environment, NF-κB and iNOS mRNA expression is increased in HCFs but not in KC-HCFs. One could speculate that KC-HCFs do not have more capacity to increase NF-κB and iNOS expression.

It might also be possible that KC-HCFs counteract a higher expression of NF-κB and iNOS to avoid an even higher mRNA concentration of iNOS.

The hypoxia-inducible factor (HIF) is a key regulator of oxygen consumption and responsible for the induction of several genes. Under hypoxic conditions, HIF is stabilized by prolyl-hydroxylases (PHDs). The HIF-α translocates into the nucleus and binds to the HIF-β unit and serves as a transcription factor for several genes, e.g., for iNOS.

It is quite common to use different times of hypoxic exposure to simulate acute, prolonged, or even chronic hypoxia [25].

Acute hypoxia (4 h) may have a different effect on the activation of HIF, NF-kB, and iNOS than continuous (24 h) or chronic (48 h) hypoxic induction. Therefore, we chose different hypoxic exposure times, in order to detect a possible difference between responses of normal and KC cells. The HIF-1α and HIF-2α subunits are similar in function, but are expressed differently in different tissues. Although the experiments did not show differences in HIF-1α and HIF-2α mRNA and protein expression in KC and normal HCFs, as half-life time of HIF proteins is still extremely short (4–6 min) under hypoxia, we could also have missed the presence of the HIF proteins during our measurement series [26]. Likewise, the expression of HIF-1α and HIF-2α seems to be regulated in the same way in normal and KC-HCFs.

The explanation could be that the iNOS regulation is not triggered by HIF, but is regulated directly by NF-κB. The homodimer NF-κp65 (which was measured in our present study as mRNA and protein) can bind directly to the iNOS gene promoter to regulate iNOS expression in myeloid cells [27], and a similar regulatory mechanism might exist in keratoconus cells (Fig. 1). This theory is supported by the fact that PHD2 mRNA and protein expression is similar in HCFs and KC-HCFs after 48 h, although 44 h earlier, PHD2 mRNA is still in increased in normal HCFs and decreased in KC-HCFs (after 4 h). This behavior may be explained by the metabolic dysfunction due to oxidative stress in KC cells.

It is known that the ROS level of keratoconus cells is higher than in healthy cells. Our studies are consistent with the experiments of Chwa et al., who also reported higher ROS activity of KC-HCFs compared with normal controls [5]. However, there are no studies on the effect of hypoxia on ROS production in corneal fibroblasts. In our experiments, we could show that ROS concentration was lower in HCFs and KC-HCFs after induction of a hypoxic environment.

Hypoxia in glioma cells has been shown to increase the production of free radicals, notably ROS [28]. While cellular responses to hypoxia have been studied extensively, it remains controversial, whether ROS levels increase or decrease at low O_2_, depending on cell type [29].

In our case, corneal fibroblasts behaved similar to dermal fibroblasts, with a decrease of ROS concentration following hypoxia [29].

Oxidative stress may also regulate the proliferation rate of some cells. The proliferation rate of KC-HCFs was reduced compared with normal cells. Since several proteins like adaptor-related protein complex 2 (AP2B1) and immunoglobulin Lambda-like polypeptide 1(IGLL1), cell division cycle, and apoptosis regulator 1 (CCAR1) are altered in KC, there may be an altered proliferation and apoptosis-related program of these cells [30]. Our experiments show that the proliferation rate after hypoxia increases only in KC-HCFs, but not in normal control cells. It could be a counter-regulation to increase the proliferation of cells, since in keratoconus there is a reduced cell density in the stroma. Nevertheless, future studies have to clarify the exact proliferation-related alterations in KC-HCFs.

Many of the known cell physiological changes in keratoconus can be associated with inflammation, oxidative stress, and hypoxia. Our experiments suggest that the response of normal and KC human fibroblasts to a hypoxic environment may differ to some extent.

## Conclusion

In summary, a hypoxic environment increases NF-κB and iNOS mRNA expression in normal HCFs but not in KC-HCFs. It is possible that the already existing oxidative stress in KC cells leads to an altered regulation of these parameters, or that the cells no longer have the capacity to further increase NF-κB and iNOS mRNA due to the metabolic changes.

NF-κB regulation is different in normal and KC cells; it can be speculated that KC cells need longer time to normalize the NF-kB protein following hypoxia, due to the elevated stress level of these cells.

HIF and PHD2 show no altered iNOS regulation under hypoxic conditions in KC-HCFs and normal HCF, so they do not seem to play a role in keratoconus pathogenesis.

Increased cell proliferation may indicate compensatory mechanisms under hypoxia in KC.

Further studies should investigate the trigger mechanism of NF-κB and the binding of the homodimer NF-κB p65 to the iNOS promoter of corneal fibroblasts.

The relationship of an inflammatory component to oxidative stress should be further investigated, to better understand the pathogenesis of the disease.

## Data Availability

The data used to support the findings of this study are available from the corresponding author upon request.

## Abbreviations

KC: keratoconus
iNOS: inducible nitric oxide synthase
HIF: hypoxia-inducible factor
PHD2: hypoxia-inducible factor prolyl-hydroxylase 2
ROS: reactive oxygen species
HCF: human corneal fibroblast
KC-HCF: keratoconus human corneal fibroblast
NF-κB: nuclear factor kappa B
IL-1: interleukin-1
TNF-α: tumor necrosis factor alpha
IFN-γ: interferon gamma
LPS: lipopolysaccharide
CoCl2: cobalt chloride
